# “People are more afraid of a dementia diagnosis than of death”: The challenges of supporting advance care planning for persons with dementia in community settings

**DOI:** 10.3389/frdem.2022.1043661

**Published:** 2022-11-02

**Authors:** Tamara Sussman, Bianca Tétrault

**Affiliations:** School of Social Work, McGill University, Montreal, QC, Canada

**Keywords:** Alzheimer's disease, dementia care, community-based settings, advance care planning (ACP), end-of-life (EOL), reflective debriefing, non-medical settings

## Abstract

Improving early uptake of advance care planning (ACP) for persons with dementia and their families requires that staff in community-based settings feel armed and equipped to encourage and support this process. Yet few studies have explored whether staff within non-medical environments feel prepared to support early ACP engagement for persons with early-stage dementia and their families. Our qualitative interpretivist study aimed to fill this gap by facilitating, transcribing and thematically analyzing deliberations from three focus groups with 17 community-based staff. Our findings revealed four key barriers to ACP activation in community settings: (1) the stigma associated with the condition; (2) lack of knowledge about end-of-life concerns for persons with dementia; (3) uncertainties about managing complex family dynamics and (4) worries that opening up conversations about future care may lead to the expression of wishes that could not be actualized (e.g., dying at home). Our findings further revealed that ACP engagement was facilitated when staff expressed confidence in their capacities to gauge readiness, viewed themselves as guides rather than experts and had access to resources to supplement their knowledge. Reflexive training opportunities and access to materials and resources around end-of-life care for persons with dementia, could equip staff in these non-medical settings with the skills to engage in ongoing dialogue about future care issues with persons living with dementia and their families.

## Introduction

Globally, the number of people living and dying with dementia is rising (Prince et al., 2016). The estimated 55 million persons living with dementia in 2019 is expected to almost triple by 2050 (World Health Organization, 2021). Hence, practices that promise to improve people with dementia's quality of life and quality of care are sorely needed. Advance care planning (ACP) is one such practice as it places persons with chronic and progressive conditions at the center of their own future care planning by encouraging them to reflect on, communicate and if desirable document their personal values and preferences for future care in advance of need (World Health Organization World Bank, 2011; Cognitive Decline Partnership Centre, 2016; Sudore et al., 2017; Wendrich-van Dael et al., 2020). While the ideal timing of ACP is still the subject of much debate, most agree that ACP should be activated early, when persons with dementia are most consistently able to express their future care wishes and concerns to family/close friends, legally appointed decision-makers and health providers (Robinson et al., 2010; Lee et al., 2017; Piers et al., 2018; Dening et al., 2019; Huang et al., 2020; Kaasalainen et al., 2021).

In an effort to improve early ACP uptake for all adults regardless of illness status, public awareness campaigns such as *Advance Care Planning Day in Canada* (Canadian Hospice Palliative Care Association, 2021) and National Advance Care Planning Week in Australia (Advance Care Planning Australia, 2021) are beginning to emerge. These initiatives ensure public access to materials that support ACP by providing free on-line resources such as those developed by, “The Conversation Project” (The Conversation Project and Institute for Healthcare Improvement, 2016) and “The Speak Up Campaign” (Howard et al., 2020). The emergence of public materials and campaigns are reflective of a global call for “grief literacy” which positions discussions about death and dying as everyone's responsibility and suggests that reserving such discussions to medical settings results in the silencing of meaningful exchanges and connections (Tompkins, 2018; Breen et al., 2020; Kellehear, 2020).

Despite these growing efforts to normalize end of life conversations and encourage all adults to engage in future planning, these self-directed ACP resources are underutilized by persons with dementia and their families (Ryan et al., 2017; Jeong et al., 2019; Breen et al., 2020; Kellehear, 2020; Stajduhar, 2020). This leaves the responsibility of ACP activation for persons with dementia to remain with medical professionals (Dixon and Knapp, 2018; Bally et al., 2019; Bernard et al., 2020). Yet when initiated within medical settings ACP is often rushed, formalized, and focused on preferred medical treatments the precise barriers such public programs have attempted to mitigate (Prince et al., 2016; Gilissen et al., 2017; Nedjat-Haiem et al., 2017; Ryan et al., 2017; Batchelor et al., 2019; Dening et al., 2019).

Community-based settings that offer social and emotional support and programming may offer a promising alternative for improving ACP uptake for persons with dementia and their families (Seymour et al., 2010, 2013; Blackford and Street, 2012; Gardiner et al., 2012; Nedjat-Haiem et al., 2017; Prince-Paul and DiFranco, 2017; Batchelor et al., 2019; Selman et al., 2020; Nouri et al., 2021). These settings, which are largely staffed by non-medical professionals such as educators, recreational therapists, counselors and social service workers focus on whole person care by providing social and emotional programming to older persons and their families (Calista and Tjia, 2017; Litzelman et al., 2017; Nedjat-Haiem et al., 2017; Tompkins, 2018; Kellehear, 2020; Wendrich-van Dael et al., 2020; Nouri et al., 2021). As such these organizations can be considered sites of care that hold promise in the promotion of compassionate communities and cities.

Although a literature on the potential for ACP engagement in these community environments is beginning to emerge, few studies have explored whether staff within these environments, feel equipped to support early ACP engagement for persons with dementia and their families (Blackford and Street, 2012; Seymour et al., 2013; Litzelman et al., 2017; Nedjat-Haiem et al., 2017; Batchelor et al., 2019; Jeong et al., 2019; Kelly et al., 2019). Gaining an understanding of the extent to which staff in these care environments feel equipped and ready to introduce ACP to persons with dementia and their families is both warranted and timely (Dixon and Knapp, 2018; Bally et al., 2019; Kelly et al., 2019; Manthorpe and Goodwin, 2019; Selman et al., 2020; Sussman et al., 2020).

This study aims to contribute to the literature on ACP activation and uptake for persons with dementia by exploring (1) how, if at all, staff in community-based settings support ACP engagement for persons with dementia (2) what role, if any, staff in these environments feel equipped to play in ACP activation for persons with dementia and their families and finally (3) what staff feel it would take to improve ACP uptake for persons with dementia and their families in these environments.

## Materials and methods

We used an interpretive descriptive approach informed by the principles of reflexive thematic analysis to explore and inductively analyze participants' experiences (Thorne, 2016; Braun et al., 2019). This constructivist approach encourages researchers to attend to and use their own expertise to develop rich and meaningful interpretations of the data that can be used to guide practice (Thorne, 2016). This approach provided us with avenues for applying our collective expertise in dementia, community practice and end of life communication to the research process. We selected focus groups as a method of data collection because participants can help one another elaborate upon, and exchange ideas and perspectives on shared practice issues (Krueger and Casey, 2014).

This research study was conducted in accordance with the standards of the Tri-Council Policy Statement: Ethical Conduct for Research Involving Humans and was approved by the Research Ethics Board Offices at McGill University, McMaster University and at the affiliated Integrated University Health and Social Service Center.

### Site selection and recruitment

Little direction is offered on the number of focus groups required to answer a research question (Carlsen and Glenton, 2011). We elected to focus our energies on organizing three rich focus groups that represented service providers who were connected with the range of community services typically accessed by older persons with dementia and their families in the area of study. We recruited staff from two Alzheimer Society chapters (one in Ontario and one in Québec, Canada) and one home care program affiliated with a health and social services center (HSSC) in Québec, Canada. One administrator in each organization assisted with recruitment by inviting the research team to meetings attended by staff charged with providing educational support and guidance to persons with dementia and families in their respective organizations. Staff interested in learning more about the study provided their name and contact information to the research team who followed up by email or telephone with more information about the study and the date and time of the focus group. Administrative staff endorsed the project by permitting staff to participate in focus group deliberations during paid working hours.

The research team shared information with approximately fifty staff across all three settings. Twenty-three staff expressed an initial interest in participating of which 17 consented and participated in one of three focus groups (the focus groups had five, four, and eight participants, respectively). Participants in the Alzheimer Society groups were social workers and educators/counselors. Participants in the home care group were social workers and nurses. Six of seventeen participants had received some form of ACP training within the last year.

### Data collection

Two members of the research team co-facilitated each 60–90 min focus group. We developed a semi-structured interview guide to frame the focus groups discussions. At the start of each focus group, we provided a definition of ACP that emphasized its process orientation (thinking about, talking about, and occasionally documenting); holistic nature (social, emotional and spiritual elements in addition to medical preferences); and purpose (to identify and communicate care preferences and values for future care in advance of need). We offered this holistic definition so that we could ensure a common understanding prior to exploring how ACP was, or was not, being applied in practice (Givens et al., 2018; Phenwan et al., 2020; Wendrich-van Dael et al., 2020). Focus group participants were then asked to share their thoughts and experiences on (1) when if at all they engage in these discussions with persons with dementia and their families, (2) how they feel about having such conversations, and (3) the benefits and challenges of activating ACP engagement in the context of their interactions with persons with dementia and their families. Achieving saturation was an important component of data collection given the data were derived from three focus groups. Hence probes, and additional questioning were used, as needed, to ensure that a full understanding of participants' perceptions and experiences was achieved (Legard et al., 2003). We obtained written consent from all participants prior to conducting each focus group.

### Data analysis

We audio-recorded, transcribed and thematically analyzed the focus group deliberations in six stages (Braun et al., 2019). In the first stage, we read the focus group transcripts independently, documenting our observations and possible meanings in the margin of the text. We then discussed these observations and identified initial codes we thought broadly captured participants' thoughts, experiences, and reactions to ACP engagement with persons with dementia (Marshall and Rossman, 2015). We developed descriptive codes such as *challenges related to staff engaging in ACP* and *resources to support staff members' engagement with ACP* at this stage. In the second stage, the second author matched initial codes with extracts from transcripts (Kidd and Parshall, 2000). We used large parts of extracts (typically two paragraphs) to ensure the context was preserved. In the third stage we reviewed coded extracts and discussed possible meanings and patterns within, between, and across codes to develop tentative categories (Marshall and Rossman, 2015). For example, at this stage we noted that stigma and limited end of life (EOL) knowledge emerged as key challenges to staff engagement with ACP. We hence developed preliminary categories such as *Internalized Stigma* and *Limited EOL knowledge* and explored how, when, and in what way these issues emerged as barriers to ACP engagement. In the fourth stage, we went back to the original transcripts to develop a more detailed analysis of the factors emerging as challenges to ACP engagement looking for accuracy and redundancy within and between categories (Marshall and Rossman, 2015). This stage also involved a focused exploration of the sentiments purported to support staff members' engagement with ACP across settings and create a comprehensive account of staffs' experiences of ACP within and across settings (Marshall and Rossman, 2015). In the fifth stage, we reviewed emergent categories extensively and continued to refine and rename them so that the scope and focus of each was clear. This led to the development of a series of themes that more clearly connected our observations of differential practices to ACP engagement and also centered the concept of staff role/position as a critical condition for engaging in ACP discussions. Finally, in the sixth stage we wrote and refined the written formulation of the findings. At this stage, all coded French extracts were translated by the second author and verified for accuracy by the first author. The team's capacity to work with French transcripts until the final stage of analysis aligns with recommendations in the literature, as it helps to preserve the contextual meanings of extracted text (Roth, 2013). At this last stage we also assigned all participants pseudonyms to maintain confidentiality while allowing their personal thoughts and experiences to be followed.

## Results

While all participants agreed that they or their organizations had some role to play in supporting ACP engagement for persons with dementia, hesitations prevailed amongst participants in both homecare and community care settings. Factors that appeared to inhibit ACP engagement among participants across sites included the stigma associated with the condition, a lack of knowledge about end-of-life concerns for persons with dementia, uncertainties about managing complex family dynamics and worries that opening up conversations about future care may lead to the expression of wishes that could not be actualized (e.g., dying at home). Factors that appeared to facilitate ACP engagement included confidence in gauging readiness through attunement, positioning oneself as a guide and having access to resources such as an interdisciplinary team to supplement knowledge. The findings presented below highlight the way in which these factors could work together to challenge or support ACP engagement in community settings.

### Theme one: Dementia stigma impacts staff [d]iscomfort with ACP engagement

While all participants provided initial endorsement for the importance of activating ACP conversations with persons with dementia, focus group deliberations quickly revealed many hesitations on the part of staff who considered the topic “very touchy” (Kai) because of the negative connotations associated with advanced stages of dementia. As one participant stated, “the only thing worse than [receiving a diagnosis of dementia] is the idea of going to a nursing home” (Alex). Another added “people are more afraid of a dementia diagnosis than dying” (Kai). Seen in this regard, inviting conversations about ACP meant placing clients in the precarious position of accepting a highly stigmatized label.

Although staff framed stigma as a barrier impacting client and family readiness, focus group deliberations further revealed that for some staff, internalized negative beliefs about the condition challenged them to consider ACP as a viable and useful practice. In these instances, staff themselves described dementia as a dehumanizing and undignified condition and hence questioned the utility of encouraging persons with dementia and families to think about the future:

The thought of me walking around with incontinence products, my shirt on backwards […] I think for our population it is an incredibly difficult conversation […] I think the people with dementia are overwhelmed enough by their diagnosis and dealing with what the diagnosis of dementia means. And I think that's scary enough for them to think about the future with. (Drew)

When staff had their own internalized stigma associated with the condition, they tended to understand and accept why some persons with dementia would deny the reality of their condition and may “never be ready to engage in the[se] discussion[s]” (Ezra). Conversely staff whose personal feelings about dementia were less apparent, saw navigating through denial as a complex yet important step in tackling ACP engagement for persons with dementia. As one home care staff stated “my experience has been that it's very difficult because the starting point is often denial. There's an inability to put [a] finger on what's going wrong so that getting past that to the kind of discussions that we would hope to have [...] there's a few steps and even then, they're not easy and we don't always get there...” (Cameron)

Stigmatized notions of dementia also emerged more subtly through discussions of capacity. For example, although focus group discussions asked staff to consider ACP engagement with persons with early-stage dementia, participants suggested that there is “always the question of the lucidity” (Cameron) when considering ACP engagement with persons with dementia. In fact, staff in home care environments suggested that by the time persons with dementia sought services “the[ir] dementia [was] pretty far gone …[and]…it [was] impossible to get […] answers from the client that [were] coherent […]” (Kris). In most of these instances, staff did not approach ACP as they presumed persons with dementia were not positioned to speak to their future care wishes and preferences.

Social stereotypes about older adults and aging also appeared to impede the ACP process. This form of stigma emerged when staff suggested it was easier to discuss ACP with younger persons experiencing early onset dementia than with other persons with dementia that are older and hence less amenable to such discussions. These sentiments were expressed as follows:

[…] Maybe because of the generation, so when I speak to these [older] clients or their spouses or their caregivers about, you know, roles and mandates and all that, they don't want to talk. Half the time they don't want to talk about it. They don't want to plan [for the] future. They're not interested. But the next generation with early-onset dementia, people in their forties and fifties, are more willing to discuss those things [and] are more willing to talk about the future. They're more interested in future planning in my experiences. (Kris)

In sum, while staff across focus groups suggested that ACP was an important component of care planning, the stigma associated with the condition (deterioration and incapacity) appeared to deter staff from broaching the subject. Staff with their own internalized sense of stigma or concerns about capacity appeared most reticent to engage in ACP discussions wondering if such conversations were even possible or would do more harm than good.

### Theme two: Familiarity with end-of-life and dementia care needs supports staff engagement in ACP

While all staff had a general sense of comfort discussing how current losses have been impacting persons with dementia, their hesitations discussing future care were exacerbated by a lack of knowledge regarding the issues and concerns typically faced by persons with dementia at end of life. This was particularly true of staff in Alzheimer Society settings whose staffing consisted of educators, counselors and social workers.

…. I don't know that we're always prepared to answer those questions. It's difficult to keep up. We don't always have, uh, someone with knowledge of that like a nurse who works in palliative care. We don't always have access to that. (Jaylin)

Not knowing what care decisions may lie ahead for persons with end stage dementia made it difficult for staff to help persons with dementia reflect on areas of importance.

[...] I don't think we necessarily have the tools on how to explain what's going on. What are the situations [that will happen] or [if] the decisions we make will have consequences, etc. (Taylor)

The lack of knowledge and comfort regarding issues of importance meant for some that even when persons with dementia who were ready to engage in open discussions and who sought support in doing so, were met with uncertainty and resistance from staff as reflected in the following statement:

I think that what she has just said, [...] the instant we open the subject [discussion] we don't have the impression that we are closing it directly because, even us, I don't think we are equipped to know this mandate. For this type of mandate, you have to refer to [a specific] person, [like to their] doctor [...]. I think that's it [and] unlike my colleagues, I'm [...] a counselor and I've had situations where people were really ready to talk about it [ACP] and it was like [...] where do I start? You know, it seems like [even] if that door is open, we're not ready I think. (Taylor)

Importantly, this theme largely emerged in the Alzheimer's Society group where medical practitioners are rarely if ever employed or accessible.

### Theme three: Unrealistic directives and family disagreements made ACP challenging to navigate

There were two very common areas staff across settings and disciplinary backgrounds regarded as key barriers to moving forward with advance care planning: navigating expressed future wishes considered unrealistic and managing families with conflicting views and sentiments. For example, staff suggested it was common for persons with dementia to direct families to “Never put [them] into one of those nursing homes.” (Ezra)

In these cases, staff felt uncertain about how to navigate a future care conversation as they did not want to give the mistaken impression that this outcome could be avoided. As one home care staff stated “Sometimes it's hard. The person is lucid, she has all her wits about her and she has a will, she wants to stay at home. But sometimes, it's difficult to work with this kind of person [who wants to stay home but who cannot].” (Claude)

In cases where divergent views between an individual and their family occur, staff in all settings felt unsure how to mediate the tension and opposition while at the same time respecting the choices and voice of the person with dementia –cutting short discussion and engagement in these instances. As two participants stated:

It's too bad, you know, in the sense that the person expresses their wishes and then the family is against their wishes. [...] I mean you're, there's no one better [who] knows what you want than you. (Claude)

Even sometimes in treatment [...] they [person with dementia] don't want a treatment and then the family wants it. It's really difficult. Especially if the person is lucid. As we were saying earlier, how to respect the wishes of the client. Sometimes the children [of the person with dementia] get involved and it becomes difficult when the client is lucid too. (Elie)

Particularly divisive topics identified by staff that fuel family disagreement included divergent sentiments around life-sustaining treatments and when persons with dementia expressed a desire to die rather than live with particular deficiencies. One home care staff commenting on decisions around life sustaining treatments expressed the challenges as follows:

[For] me [...] what I find particularly difficult sometimes is [in the case] when the sick person [...] including [persons with] dementia they want to share their wishes with their children, for example. Oh-la-la. Sometimes it's great because the values coincide. If you can call it that. But sometimes, uh, it's a crisis. [For example] ”Mom, why do you want this? Do you want to die¿‘ Because the mother doesn't want to be on the ventilator. Let's face it [...] I find it a challenge for the person to share their wishes with their children, with their family. And then me, to be there to try to facilitate the conversation. Sometimes I'm like, ”I want to leave.“ (Dominique)

Another staff person who spoke of the complexities when a person with dementia speaks of a desire to die as follows: “I find myself in the middle. I see both sides [talking about suicide] but then I don't feel equipped to do it [have the conversation].” (Jan)

These latter conversations were viewed as more common in the last 2 years with the advent of Medical Assistance in Dying.

In sum, sentiments expressed within and across groups suggested that many staff felt ill equipped to navigate ACP conversations when unrealistic wishes were expressed by persons with dementia or when disagreements emerged between family members about managing end of life issues including accessing life sustaining treatments and or expediting death. In these instances, staff described feeling ineffective, uncertain of how and where to take the conversation and hesitant to re-introduce or revisit ACP conversations.

### Theme four: Overcoming barriers of ACP engagement through positionality, attunement and having access to resources

While staff expressed a range of challenges both initiating and sustaining ACP engagement, positioning oneself as a guide rather than an expert, staying attuned to the emotional reactions of persons with dementia and their families and having access to clinical expertise or resources appeared to help staff to engage in ACP dialogues that were viewed as useful.

One staff speaking of the importance of positioning stated:

I really like in social work the idea of accompaniment [which] is not talked about too much [...]. Like the Shaman concept [...]. (T)he idea of accompaniment [is one] where we can develop our empathy, and [when we] approach someone we have to think about, [passing] from one phase to another, that is difficult to get through. To accept, to adapt to the losses of autonomy, to adapt to the decrease in functioning. To adapt to new medical instructions. Adapting to a diagnosis - that is difficult to digest. It forces us to face human existentialism. (Cameron)

Taking and sustaining this type of position was viewed as active as it required staying attuned to reactions and adapting conversations accordingly. When seen in this light, staff appeared to appreciate the ebbs and flows of difficult ACP conversations and adjust themselves and their approach accordingly. This fluidity was expressed as follows:

[...] I personally don't feel uncomfortable talking about all the subjects. I'll go slowly. I'll see how far they go in answering. If it makes them uncomfortable, and I see that they are reluctant to talk about it, I'll maybe stop the subject for a while and then eventually we'll come back to [if] we're not there [...]. But [if I have concerns] before we're there, I will broach the subject if I have concerns [...] but if there aren't any, I don't really have any concerns about it, I'll wait until the family talks about it [...]. (Claude)

Seemingly this notion of attunement helped to alleviate stress related to the correct timing of ACP conversations because it allowed staff to slowly assess and respond to readiness. As one Alzheimer's Society staff shared, “[y]ou have to adjust yourself to realize […] if they're ready [or not] for that conversation, if [they're] not, [it's] not going to go anywhere […] you have to pull back 'cause that's not being person-centered.” (Kai)

However, when it came to the management of family disagreements or unrealistic wishes these skills did little to arm staff with the comfort and confidence they required to support ACP dialogue.

Having access to resources “clinical supervision” and specialized knowledge from an interdisciplinary team also appeared to provide staff with the confidence and comfort needed to initiate ACP conversations because staff knew they had a place to go to reflect on their uncertainties particularly pertaining to EOL knowledge and information. As two participants stated:

We were just talking about it last week in supervision, it's hard to know the link between I understand why he would want to kill [himself] 1 day, but we are in a quality of life approach [in our work] [...]. (Jan)

When we confront, you know, either ethical difficulties or other complications, we discuss it with our supervisors or the spécialiste en clinique (clinical specialist). (Cameron)

When these standpoints, skills and resources were unavailable to staff they preferred that these conversations were “[….] done at the doctor's office” (Drew) as they did not feel positioned or equipped to manage them.

## Discussion

Advance care planning (ACP) encourages persons with chronic and progressive conditions to reflect on, communicate and if desirable document their personal values and preferences for future care in advance of need (Sudore et al., 2017). Improving early uptake of ACP for persons with dementia and their families requires that staff across the trajectory of care feel armed and equipped to encourage and support this process. This is particularly pertinent for community-based staff who have been identified as key sources of support for persons with dementia and their families (Morton-Chang et al., 2016; Chan et al., 2020; O'Connor et al., 2022). Yet, as our findings illuminate, much more needs to be done to equip staff in these non-medical settings to initiate and encourage ongoing dialogue about future care issues with persons living with dementia and their families.

While knowledge related to end-of-life care for persons with dementia emerged as a deterrent for some community-based staff, stigma associated with the condition appeared to play a critical role in staff's willingness to broach the topic of future care with persons with dementia and their families. More specifically, staff who associated a future with dementia solely around loss, deterioration and incapacity, wondered when, if at all, it would be of utility to encourage future care reflections with persons with dementia and their families (Prince et al., 2016). Viewing dementia in this way meant that thinking about the future would conjure a sense of bleakness that would evoke strong emotional reactions such as fear, anxiety, and distress (Gilissen et al., 2017; Nedjat-Haiem et al., 2017; Kim et al., 2019; Wendrich-van Dael et al., 2020). This finding affirms trends noted in the literature regarding the prevalence of stigma among staff in community settings (Herrmann et al., 2019; Kim et al., 2019; Wendrich-van Dael et al., 2020) and the impact this can have on broaching conversations about the condition including but not limited to ACP engagement (Batsch and Mittelman, 2012; Benbow and Jolley, 2012; Dening et al., 2019; Chan et al., 2020; Van Rickstal et al., 2022). That staff in these non-medically focused settings still held very biomedically oriented views (i.e., dementia solely as physical and cognitive deterioration) serves as a strong reminder that notions of personhood and citizenship emerging as important counter narratives to what living with dementia can and does mean are still far from prevalent (Kitwood, 1997; Bartlett and O'Connor, 2010; Kontos and Martin, 2013; Foth et al., 2016). Our study further highlighted the ways in which ageism and assumptions around incapacity could work together to inhibit staff from initiating ACP conversations with persons with dementia and their families (Barber, 2017; Lynch, 2020; Hassan, 2021). More specifically, some staff presumed older persons would be less inclined to engage in future care conversations than younger persons and others suggested that capacity should always be questioned when working with persons with dementia. This stance stands in stark contrast with article 12 of the United Convention on the Rights of Persons with Disabilities (United Nations General Assembly, 2006) and the *Handbook for Parliamentarians on the Convention on the Rights of Persons with Disabilities* (Mason and Munn-Rivard, 2021) which frame capacity as a human right and cautions professions to presume rather than question capacity in most circumstances and helps to keep Canada and other countries on track in upholding and advancing the rights of people with disabilities. Taken together these sentiments suggest that older persons with dementia and persons showing some evidence of cognitive decline may be at heightened risk of exclusion from ACP conversations amongst community-based staff.

On a more positive note, staff who appreciated that persons living with dementia could grow, evolve and change and who were confident in their capacity to position themselves as guides appeared more confident initiating ACP discussions as they knew they could retract and redirect their questions as needed without doing irreparable harm. These staff appreciated that an invitation to discuss ACP related issues such as fears and concerns about future care was the first step in identifying a “right time” for such conversations. They also understood that relational connections were more important than knowledge and rank when inviting difficult conversations about ACP (Tilburgs et al., 2018). However, even those workers felt immobilized in instances when there was disagreement amongst family members, when persons with dementia expressed unrealistic care preferences, when assisted suicide was brought up and/or when they lacked the back-up resources or information they needed to follow up on specific questions and concerns they could not address themselves. These insights offer some important directions for future training and initiatives needed to improve the capacity of community settings to play a role in encouraging and supporting ACP conversations with persons with dementia and their families.

### Implications and recommendations

Training opportunities such as team huddles, comfort care rounds or reflective debriefing programs, may offer promise in improving community-based staff's capacities for ACP engagement with persons with dementia (Hockley, 2014). Typically facilitated by a clinical expert or consultant, these opportunities enhance peer-based support, provide in-the-moment education, and help translate principles into the real world of practice (Seymour et al., 2013; Browning and Cruz, 2018; Kaasalainen et al., 2019). Such initiatives may provide opportunities to address internalized stigma, discuss complex case materials, and provide the periodic resource support considered useful in bolstering comfort. For example, our focus group deliberations suggested that staff find it difficult to navigate ACP when potentially unrealistic plans are expressed such as when a person with dementia says, “I never want to be placed in a nursing home.” Rather than conclude that ACP is impossible in such circumstances staff can be trained to engage in a deeper discussion about the principles behind such a plan by asking questions such as “what is it about staying home that is so important to you?” Examining the extent to which such initiatives can address the barriers to ACP engagement noted above would be warranted (Herrmann et al., 2019).

Resources such as the Comfort Care Booklet for Persons with Dementia (Bavelaar et al., 2022) may also be useful for staff in community-based settings as they include issues typically faced by persons with dementia and their families at end of life. However, as our findings suggest, improving accessibility to this form of knowledge may be a necessary but not sufficient step toward improving community-based staffs' comfort with ACP activation and support.

Peer facilitated ACP workshops that use real stories to describe the value and importance of ACP conversations may help community-based settings introduce ACP to persons with dementia and their families (Sanders et al., 2006; Clarke et al., 2009; Seymour et al., 2013; Sellars et al., 2019). However, as our findings suggest if staff in these organizations lack the capacity and comfort to follow-up on these initial workshops ongoing ACP engagement may not be sustained.

### Study limitations

Our findings should be considered in light of three limitations. First, our small sample of focus group participants were located in two provinces in Canada without provincially established processes and practices for ACP. Second, the discomfort expressed when persons with dementia brought up wishes around death and dying may be unique to our study context where medical aid in dying legislation is quite new and persons with dementia are to date excluded. Third, many of the staff included in our focus group deliberations had some professional training in social work, nursing of counseling education. These issues may limit the transferability of our findings to other jurisdictions with more established laws and practices around medical aid in dying, other regions with more prescribed ACP processes and community settings who employ workers with less formalized training.

## Conclusion

Improving early uptake of ACP for persons with dementia and their families requires that staff in community-based settings feel armed and equipped to encourage and support this process. Yet the stigma associated with the condition, lack of knowledge about end of life concerns for persons with dementia, and uncertainties about managing family dynamics and unrealistic wishes work together to impede ACP engagement in these settings. Reflexive training opportunities and access to materials and resources around end-of-life care for persons with dementia, could equip staff in these non-medical settings with the skills to engage in ongoing dialogue about future care issues with persons living with dementia and their families. Such initiatives appear sorely needed to support the development of compassionate communities and grief literacy in settings closely connected to persons with dementia and their families who employ limited to no medically trained staff.

## Data availability statement

The raw data supporting the conclusions of this article will be made available by the authors, without undue reservation.

## Ethics statement

The studies involving human participants were reviewed and approved by the Office of Research Ethics Boards at McGill and McMaster University. The patients/participants provided their written informed consent to participate in this study.

## Author contributions

TS conceptualized the study from which data was collected and assisted in the analysis and writing of the manuscript. BT led the analysis and co-wrote the manuscript. Both authors contributed equally to the writing of the article. Both authors contributed to the article and approved the submitted version.

## Funding

This work was supported by funding from the Alzheimer Society of Canada Quality of Life Research Grant 2017 – 2020 [18 – 19].

## Conflict of interest

The authors declare that the research was conducted in the absence of any commercial or financial relationships that could be construed as a potential conflict of interest.

## Publisher's note

All claims expressed in this article are solely those of the authors and do not necessarily represent those of their affiliated organizations, or those of the publisher, the editors and the reviewers. Any product that may be evaluated in this article, or claim that may be made by its manufacturer, is not guaranteed or endorsed by the publisher.
